# A Novel Microfluidic-Based OMC-PEDOT-PSS Composite Electrochemical Sensor for Continuous Dopamine Monitoring

**DOI:** 10.3390/bios13010068

**Published:** 2022-12-31

**Authors:** Sofwan Nuh, Apon Numnuam, Panote Thavarungkul, Tonghathai Phairatana

**Affiliations:** 1 Department of Biomedical Sciences and Biomedical Engineering, Faculty of Medicine, Prince of Songkla University, Songkhla 90110, Thailand; 2 Center of Excellence for Trace Analysis and Biosensor, Prince of Songkla University, Songkhla 90110, Thailand; 3 Division of Physical Science, Faculty of Science, Prince of Songkla University, Songkhla 90110, Thailand; 4 Center of Excellence for Innovation in Chemistry, Faculty of Science, Prince of Songkla University, Songkhla 90110, Thailand; 5 Institute of Biomedical Engineering, Faculty of Medicine, Prince of Songkla University, Songkhla 90110, Thailand

**Keywords:** dopamine, amperometry, microfluidics, ordered mesoporous carbon, continuous monitoring

## Abstract

Fast and precise analysis techniques using small sample volumes are required for next-generation clinical monitoring at the patient’s bedside, so as to provide the clinician with relevant chemical data in real-time. The integration of an electrochemical sensor into a microfluidic chip allows for the achievement of real-time chemical monitoring due to the low consumption of analytes, short analysis time, low cost, and compact size. In this work, dopamine, used as a model, is an important neurotransmitter responsible for controlling various vital life functions. The aim is to develop a novel serpentine microfluidic-based electrochemical sensor, using a screen-printed electrode for continuous dopamine detection. The developed sensor employed the composite of ordered mesoporous carbon (OMC) and poly (3,4 ethylenedioxythiophene)-poly (styrene sulfonate) (PEDOT-PSS). The performance of a microfluidic, integrated with the sensor, was amperometrically evaluated using a computer-controlled microfluidic platform. The microfluidic-based dopamine sensor exhibited a sensitivity of 20.2 ± 0.6 μA μmol L^−1^, and a detection limit (LOD) of 21.6 ± 0.002 nmol L^−1^, with high selectivity. This microfluidic-based electrochemical sensor was successfully employed to determine dopamine continuously, which could overcome the problem of sensor fouling with more than 90% stability for over 24 h. This novel microfluidic sensor platform provides a powerful tool for the development of a continuous dopamine detection system for human clinical application.

## 1. Introduction

Chemical monitoring in clinical settings requires fast and precise analysis to provide clinically relevant data in real-time at the patient’s bedside, in order to support clinical decision-making. Microdialysis, as a minimally invasive sampling technique, coupled with analytical techniques, have been widely applied to carry out both clinically and experimentally in vivo measurements of the chemical change in extracellular fluid of any tissue, especially in brain monitoring [1,2]. Dopamine (DA), a catecholamine neurotransmitter, plays a crucial role in regulating renal, cardiovascular, hormonal, and brain systems. An abnormal variation in DA levels is associated with neurological disorders. A low dopamine level indicates Parkinson’s disease, depression, and schizophrenia [3,4,5]; however, a high dopamine level may cause stress cardiotoxicity, leading to rapid heart rates, hypertension, heart failure, and drug addiction [6]. The DA detection both in vivo and in vitro has attracted a lot of attention for both clinical applications and biomedical research [7]. There are several analysis methods for DA determination with excellent sensitivity and selectivity, such as spectrophotometry [8,9], chemiluminescence [10], and liquid chromatography [11]. However, these methods encounter several drawbacks, including time consumption, high cost, a complex operation, difficulty in minimizing the system, and continuously tracking chemical changes in real-time. Alternatively, electrochemical analysis has a high potential due to benefits such as high sensitivity, specificity, rapid analysis, simple operation and, in particular, the capability of on-line, continuous detection with a minimized system [12]. To achieve specific sensing for on-line dopamine monitoring, microdialysis sampling has been successfully coupled to electrochemical sensors for tracking chemical dynamic changes in real-time [13,14].

The development of ideal on-line microdialysis coupled electrochemical sensors has focused on a low internal volume for collecting samples, typically in the range of 1–5 μL min^−1^ [15], with high sensitivity, specificity, and stability. Many studies have been reported using flow injection analysis (FIA) to perform electrochemical biosensors and sensors for continuous detection [16,17,18], however there are some limitations. These include time consumption with large volume, the large equipment involved, the requirement of trained personnel to operate the system, and the difficulty in minimizing the system and achieving an on-line detection. Microfluidics as a technique for the manipulation of multi-fluids in micro-scaled channels has received great interest in the miniaturization of sensing technology within biomedical research and medical applications [19]. The on-line chemical measurement is most easily achieved using microfluidic integrated electrochemical sensors, coupled with a microdialysis probe such as drugs [20], metabolites [21] and neurochemicals [22,23]. The integration of an electrochemical sensor into a microfluidic chip allows for the achievement of continuous monitoring; this is due to low samples and reagent consumption, short-time analysis, low energy consumption, cost-effectiveness and being compact in size [24].

The focus has been on two major challenges in DA electrochemical sensors. Firstly, electrode surface fouling, on account of the adsorption of its oxidation products, leads to low stability for DA detection. Secondly, the challenge is the issue of interference caused by the high concentration of coexistence in biological samples, i.e., ascorbic acid (AA) and uric acid (UA), which can be oxidized closely to the DA oxidation potential. Therefore, this can disturb the DA signal, resulting in poor selectivity [25]. Over the past ten years, there has been growing interest in the design of electrochemical sensors for neurotransmitter detection. Many research groups have extensively studied these using various carbon-based materials such as graphene [26,27], carbon nanotubes [28,29] and ordered mesoporous carbon [30] for the enhancement of sensor performances. Among these, ordered mesoporous carbon (OMC) as a carbon material with a 3D nanoporous structure has attracted a lot of attention for electrochemical sensor fabrication. This is due to its offering properties such as excellent electron transfer, large pore volume, a high specific surface area, small pore size, chemical inertness and biocompatibility [31,32]. In particular, its porous structure provides more active sites and interfaces for interactions with analytes, which is very beneficial to adsorption-desorption and promoting mass diffusion in the sensing process [32,33]. Additionally, the use of a conducting polymer, poly (3,4 ethylenedioxythiophene)-poly (styrene sulfonate) (PEDOT-PSS) for electrochemical detection has been reported. PEDOT-PSS offers high electrical conductivity, low oxidation potential, anti-fouling properties and excellent thermal stability [34,35,36]. Regarding their properties, there have been some recent studies on OMC in combination with PEDOT-PSS for the development of electrochemical sensors and biosensors [37,38].

As there have only been a limited number of DA electrochemical detection studies under continuous analysis, the aim of this work was to be the first report of a combination of OMC and PEDOT-PSS modified screen-printed carbon electrodes (SPCE), integrated with a microfluidic system for continuous electrochemical DA monitoring. The electrochemical performances of the OMC-PEDOT-PSS composited sensor were investigated and optimized using voltammetry. The serpentine microfluidic chip was designed for integrating with the developed OMC-PEDOT-PSS/SPCE, and was fabricated using soft lithography based on polydimethylsiloxane (PDMS). To perform the applicability of the developed sensor platform for continuous DA detection, the operation of this system in vitro DA amperometric detection was demonstrated for a long period of 24 h, using an in-house computer-controlled microfluidic platform. Calibrations could be frequently performed using this automated microfluidic system over the monitoring period. Thus, the concentration of DA can be accurately determined, showing a great possibility for human clinical application.

## 2. Materials and Methods

### 2.1. Chemicals and Apparatus

Poly(3,4-ethylenedioxythiophene)-poly (styrene sulfonate) (PEDOT-PSS, 1.1% in H_2_O), Nafion (~5%), dopamine hydrochloride (DA), ascorbic acid (AA), and uric acid (UA) were from Sigma-Aldrich (St. Louis, MI, USA). Ordered mesoporous carbon (OMC) was synthesized using the method detailed in a previous report [31]. Di-sodium hydrogen orthophosphate, sodium dihydrogen orthophosphate, and sodium carbonate were from Ajax Finechem (Sydney, Australia). Methanol (RCI Labscan, Australia), isopropyl alcohol (99.5%, LOBA CHEMIE, Mumbai, India), SU-8 photoresist 3050 (MicroChem, Westborough, MA, USA), SU-8 developer (Sigma-Aldrich, St. Louis, MI, USA), and poly(dimethylsiloxane) (PDMS, Sylgard 184, Dow Corning, Midland, MI, USA) were used as received. Standard solutions of dopamine (DA) were freshly prepared in a 0.10 mol L^−1^ phosphate buffer solution (PBS), at pH 7.4 for both stationary and flow conditions. All aqueous solutions were prepared with water purified by a Milli-Q purification system (≥18 MΩ cm resistivity at 25 °C).

Electrochemical experiments were performed using a DropSens μStat400 (potentiostat/Galvanostat, Spain) and monitored by DropView software. Screen-printed carbon electrodes (SPCEs, DRP-C110DIEL) from DropSens were based on a carbon working electrode (4 mm in diameter), a silver pseudo-reference electrode, and a carbon counter electrode. The surface morphologies of modified electrodes were investigated using a scanning electron microscope (SEM, Quanta 400, Czech Republic). An autocalibration microfluidic platform was set up using LabSmith components (Livermore, CA, USA), and was controlled via µProcess software.

### 2.2. Preparation of OMC and PEDOT-PSS Modified Electrodes

A SPCE was initially pretreated to eliminate excessive organic compounds on the electrode surface by applying a constant potential of 1.2 V for 5 min in saturated sodium carbonate, rinsed with deionized water and dried with nitrogen gas. Two mg of OMC was dissolved in 1.0 mL of ethanol with 0.0050% Nafion (as a material adhesion) and ultrasonicated for 1 h to obtain a homogeneous suspension. The 50 μL OMC suspension was then mixed with the 25 μL of PEDOT-PSS and ultrasonicated for 30 s. A 4.0 μL of the OMC-PEDOT-PSS composite suspension was dropped onto the working area of the pretreated SPCE and allowed to dry for 30 min. Nafion, 3.0 μL of 2.0% was then drop-casted onto the modified SPCEs as a protection layer and allowed to dry at room temperature. For comparison, an OMC modified SPCE, and a PEDOT-PSS modified SPCE were also prepared via the same procedure, using only the OMC suspension and PEDOT-PSS. Additionally, the influence of the OMC to PEDOT-PSS ratio on the sensor performances was investigated. The five ratios of OMC: PEDOT-PSS were: 1:1, 1:2, 1:3, 2:1 and 3:1 by volume, and were used to modify SPCEs; following the same steps as mentioned above. The performance of the modified sensors was electrochemically studied and optimized in terms of their current response and sensitivity.

### 2.3. Electrochemical Measurements

The electrochemical behaviors of four modified SPCEs; i.e., bare, OMC, PEDOT-PSS, and OMC-PEDOT-PSS were investigated by cyclic voltammetry in 100 μmol L^−1^ DA in 0.10 mol L^−1^ PBS (pH 7.4). For the optimization study of OMC:PEDOT-PSS composite ratio, DA concentrations were studied at: 2.0, 5.0, 10, 20, 40, 60, 80 and 100 μmol L^−1^, using differential pulse voltammetry at a scan rate of 50 mV s^−1^; with a pulse amplitude of 25 mV in 10 mL of 0.10 mol L^−1^ PBS (pH 7.4). The change in oxidation current responses versus the concentrations of DA were plotted and compared. The condition attained the highest sensitivity (slope of the calibration plot) was then employed further in the section of on-chip experiments.

The effect of interferences was studied with the optimal modified SPCE by determining DA in the presence of ascorbic acid (AA) and uric acid (UA), which coexist in the same sample because of the AA and UA having higher concentrations than DA (in the range of 100 and 1000 times) [39]. The solutions of 0.50 mmol L^−1^ AA, 0.50 mmol L^−1^ UA, and 0.010 mmol L^−1^ DA were freshly prepared in 0.10 mol L^−1^ PBS (pH 7.4). Cyclic voltammetry was performed between −0.30 V and 0.60 V at a scan rate of 50 mV s^−1^.

### 2.4. Design, Simulation, and Fabrication of Microfluidics

**Figure 1:**
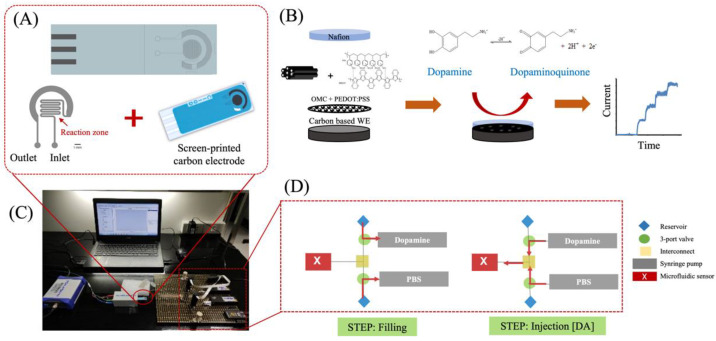
The developed microfluidic-based OMC-PEDOT-PSS sensor platform for dopamine monitoring system. (**A**) Integration of a serpentine PDMS based chip on a SPCE, serpentine pattern using Siemens NX software, (**B**) Schematic representations of the modification of OMC and PEDOT-PSS on a SPCE, (**C**) photo of the complete system of DA detection, and (**D**) the configuration of the developed computer-controlled microfluidic platform using LabSmith components.

The preliminary amperometry study of DA on an OMC-PEDOT-PSS/SPCE integrating with circle and serpentine microfluidic channels showed a more stable and a two times higher current signal of the serpentine-based OMC-PEDOT-PSS/SPCE than that of the circle-based OMC-PEDOT-PSS/SPCE (Supplementary data Figures S1 and S2); i.e., the serpentine OMC-PEDOT-PSS/SPCE can contribute to the dopamine oxidation signal. Hence, the design of a serpentine microfluidic channel for integrating with a SPCE was used. The serpentine pattern was created using Siemens NX software. It consisted of an inlet, an outlet and a reaction zone, in which the channel dimension was 100 μm in-depth, 250 μm in width and 27 mm in length (calculated in the reaction zone area; wherein the working volume of the device was 0.675 μL) (Figure 1A). The pattern was simulated using ANSYS Fluent (2019 R1), under an initial condition of 2.0 μL min^−1^ flow rate and the fluid property that was assumed to be water (Newtonian fluid; viscosity (µ) = 1.03 × 10^−3^ kg m^−1^.s^−1^, density (ρ) = 998.2 kg m^−3^, homogeneous, incompressible), which was close to the properties of the dopamine solution [40]. The total number of meshes for the serpentine pattern was 68,046 nodes, with an element size of 0.050 mm. In the fabrication process, a master mold was created using direct laser lithography (direct laser writer). Briefly, the SU-8 3050 (negative photoresist) was spin-coated on a glass substrate at 500 rpm for 10 s to form a homogeneous film, and then at 1400 rpm for 30 s to form a 100 μm thickness. The thin film-coated substrate was soft-baked at 65 °C for 5 min and increased gradually with the rate of 5 °C min^−1^ until it reached 95 °C; it was then left for 30 min. Next, a UV laser beam (375 nm, Dilase 250, KLOE) was exposed to the substrate by directly writing the designed pattern. After laser exposure, the patterned thin-film substrate was post-baked at 65 °C for 5 min, heated to 95 °C with a gradual increase of 5 °C min^−1^, and then kept at this temperature for 15 min to consolidate the pattern. Once the substrate had cooled down to an ambient temperature, the pattern was developed using a SU-8 developer, rinsed gently using isopropyl alcohol to remove the excess SU-8 photoresist and dried with nitrogen gas. To improve the adhesion of the final photoresist on the substrate, a hard-bake was performed at 150 °C for 3 min, and then left at room temperature. For the polydimethylsiloxane (PDMS)-based microfluidic chip fabrication, soft lithography was used. A PDMS prepolymer and a curing agent were mixed at a ratio of 10:0.6 by weight [41], before being poured onto the master mold. After curing in an oven at 65 °C for 2 h, the PDMS was then cut and peeled off. Both inlet and outlet holes (1.0 mm diameter) were punched before bonding for 20 s to a modified SPCE using a plasma cleaner (Harrick Plasma, New York, NY, USA). The three markers on the PDMS-based microfluidic chip were used to precisely align the modified SPCE under a stereo microscope.

### 2.5. On-Chip Experimental Setup

For continuous analysis, the optimal OMC-PEDOT-PSS modified SPCE (Figure 1B) was combined with the serpentine PDMS microfluidic channel. The complete system of an on-chip experiment is illustrated in Figure 1C. An important issue of continuous detection is sensor stability, especially under flow conditions. The performances of the developed microfluidic-based OMC-PEDOT-PSS electrochemical sensor for continuous DA measurement were investigated using our in-house automatic calibration platform. The developed platform was introduced using programmable LabSmith components that can provide excellent sequential control of multi-liquid streams with precise fluid delivery. Figure 1D schematically illustrates the calibration platform consisting of two 80 μL programmable syringe pumps (SPS01-080 LabSmith); one containing a 10 μmol L^−1^ DA standard solution and the other containing a 0.10 mol L^−1^ PBS solution (pH 7.4). Each syringe pump was connected to a valve for switching the flow direction and a reservoir containing 1.0 mL of the solution. The platform was automatically controlled via µProcess software.

To calibrate the microfluidic-based OMC-PEDOT-PSS sensor, a 5-point calibration was achieved by mixing the flows with changing relative flow rates of the syringe pumps, while keeping a constant overall flow rate of 2.0 μL min^−1^ through the microfluidic analysis system. The series of DA concentrations were generated and injected through the inlet of the developed microfluidic-based sensor (STEP: injection [DA] in Figure 1D). After each calibration cycle, the computer-controlled microfluidic platform allowed the pumps to be continually refilled (STEP: filling in Figure 1D). DA detection was via amperometry under a flow analysis system. The microfluidic-based OMC-PEDOT-PSS/SPCE, connected to a portable DropSens potentiostat, was held at a constant potential of 0.20 V as an oxidation peak of dopamine. The series of five DA concentrations; i.e., 2.0, 4.0, 6.0, 8.0, and 10 μmol L^−1^ were studied. The operational stability of the sensor was performed with the computer-controlled microfluidic platform that was used to track the performances of the developed OMC-PEDOT-PSS-based sensor for a period of 24 h (every 3 h) under continuous flow condition.

## 3. Results and Discussion

### 3.1. Surface Characterization

**Figure 2:**
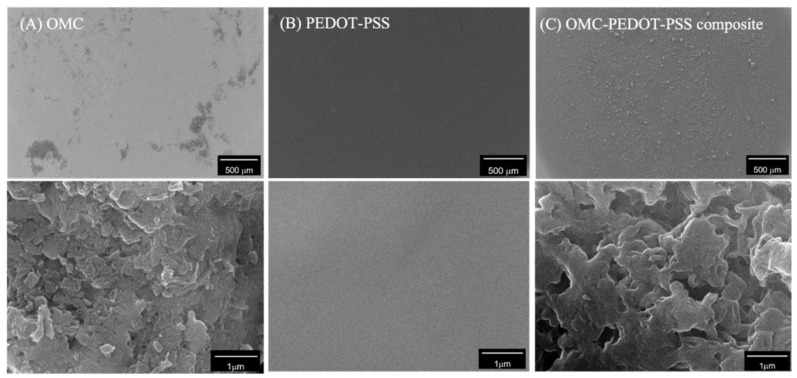
SEM images showing the morphologies of the surface modified with (**A**) OMC (**B**) PEDOT-PSS and (**C**) the composite of OMC and PEDOT-PSS.

The surface morphologies of OMC, PEDOT-PSS and the composite of OMC and PEDOT-PSS were examined using a SEM. The OMC modified electrode showed a non-uniform agglomeration on the surface (Figure 2A). These OMC particles exhibited rough and porous layers of stacked carbon plates, similar to those reported earlier [42]. For PEDOT-PSS modification, the deposition of a smooth thin uniform film was observed over the entire surface (Figure 2B). When OMC was added into PEDOT-PSS to form a composite material, the OMC-PEDOT-PSS composite was more homogeneously distributed on the surface (Figure 2C). Thus, the adding of PEDOT-PSS contributed to the uniform spreading of OMC, so as to cover the entire surface. The magnified view of the OMC-PEDOT-PSS composite surface was rough, owing to the covering of OMC by the PEDOT-PSS film.

### 3.2. Electrochemical Characterization

**Figure 3:**
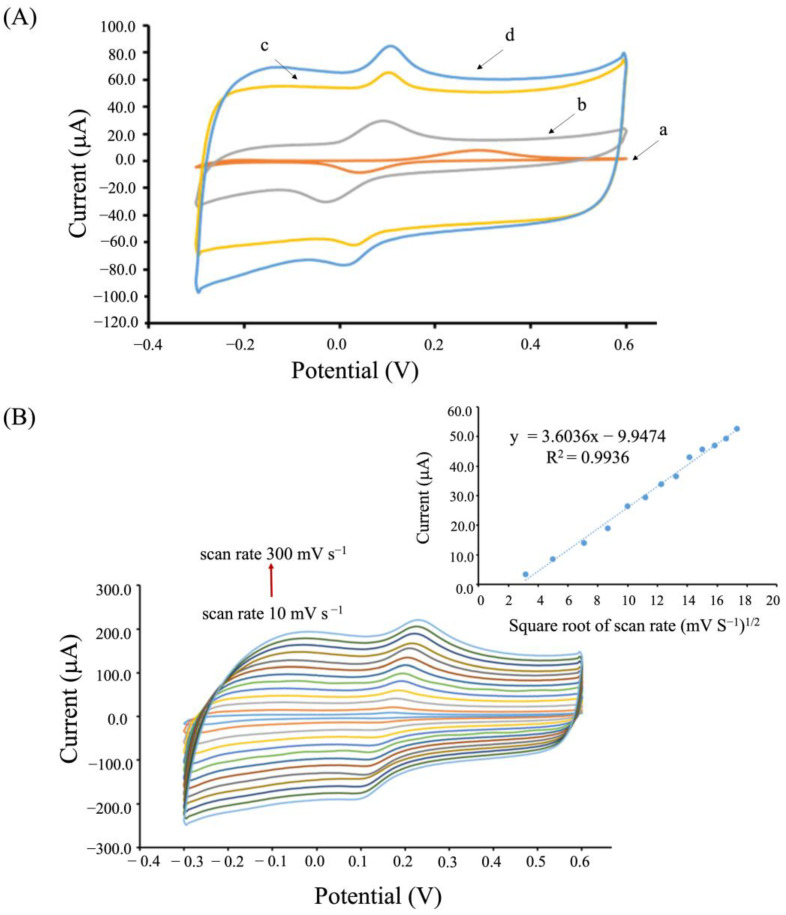
(**A**) Cyclic voltammograms (CVs) of SPCE (a), OMC/SPCE (b), PEDOT-PSS/SPCE (c), and OMC-PEDOT-PSS/SPCE (d) in 100 μmol L^−1^ DA in 0.10 mol L^−1^ PBS (pH 7.4) at a scan rate of 0.050 V s^−1^. (**B**) CVs of DA responses for OMC-PEDOT-PSS/SPCE in 100 μmol L^−1^ DA at different scan rates from 10 to 300 mV s^−1^, and the inset showing the plot between the oxidation peak currents and the square root of the scan rate.

The electrochemical behaviors of DA at each modified electrode were characterized using cyclic voltammetry, as shown in Figure 3A. At the unmodified SPCE, the cyclic voltammogram (CV) showed a pair of well-defined redox peaks (trace a); however, DA exhibited a poor electrochemical response with a broad peak potential separation (ΔEp) of 250 mV. For OMC/SPCE (trace b), it displayed better electron transfer toward DA than the unmodified SPCE, as observed from the smaller ΔEp (110 mV). This indicated that the large, active surface area and high conductivity of mesoporous materials enhanced the electrochemical activity. Additionally, the peak oxidation potential shifted negatively indicating fast electron transfer, which was in agreement with the previous report [43]. The DA signal for a PEDOT-PSS/SPCE exhibited much higher electrical conductivity than that of the unmodified SPCE, as observed from the oxidation current signal. Although the electrical conductivity of a PEDOT-PSS/SPCE was lower than that of OMC/SPCE, it provided a faster electron transfer than that of OMC/SPCE when observed from its ΔEp 90 mV. The highest oxidation current response toward DA oxidation were noticed at the OMC-PEDOT-PSS composite modified electrode, as illustrated in Figure 3A (trace d). The current response toward DA at OMC-PEDOT-PSS/SPCE was approximately 3.1, 2.3, and 1.7 times higher than that of bare SPCE, OMC/SPCE, and PEDOT-PSS/SPCE, respectively. It is likely that the rough surface, together with a homogeneous distribution of OMC on the whole electrode surface offered a large, active surface area [42] that contributed to an increase in the conductive surface area. This could result in the enhancement of the current response, and suggests that the OMC and PEDOT-PSS composite material could enhance the performance of electrochemical responses toward DA detection.

Additionally, the electrochemical behaviors of 100 μmol L^−1^ DA at different scan rates, 10 to 300 mV S^−1^, at the OMC-PEDOT-PSS/SPCE were also investigated, as shown in Figure 3B. The CVs showed an increase in the oxidation peak current and the peak potential showed a slight positive shift, with an increasing scan rate of cyclic voltammetry. This was because the electrocatalytic activity of dopamine on the OMC-PEDOT-PSS/SPCE surface was changed when decreased in the diffusion layer. The anodic peak current was proportional to the square root of the scan rate (Figure 3B inset). This result showed great linearity following the Randles-Sevcik equation, confirming that the process of dopamine oxidation at the OMC-PEDOT-PSS/SPCE occurred under diffusion-controlled reactions; i.e., electron transfer between the electrolyte and the electrode occurs close to the electrode surface (freely diffusing redox species) [44].

### 3.3. Effect of OMC: PEDOT-PSS Composite Ratios

**Figure 4:**
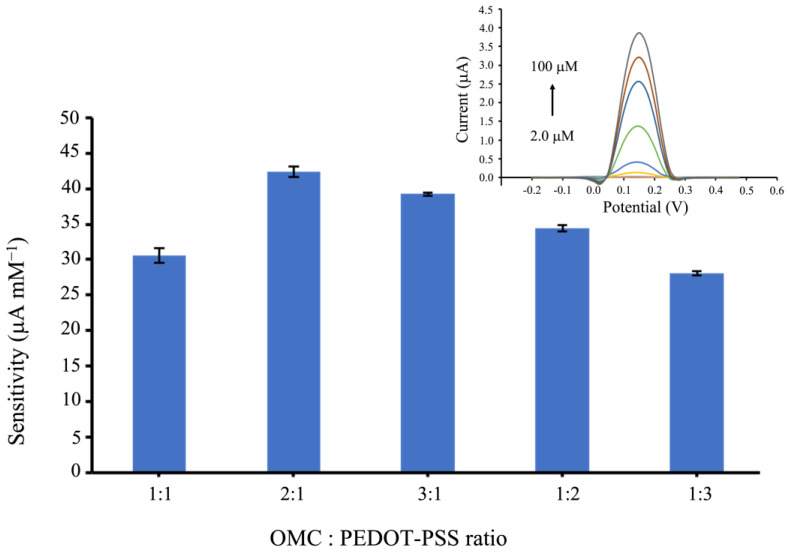
Effect on the sensitivity of the developed sensors to DA detection in the different ratios of OMC and PEDOT-PSS. These sensitivities were obtained by performing DPV in the series of DA concentrations at: 2.0, 5.0, 10, 20, 40, 60, 80, and 100 μmol L^−1^ in 0.10 mol L^−1^ PBS solution (pH 7.4). The inset showed the differential pulse voltammograms of the OMC and PEDOT-PSS at 2:1 ratio when varying from 2.0 to 100 μmol L^−1^ DA.

The ratio of OMC and PEDOT-PSS in the composite materials is a significant parameter that can affect the performance of an OMC-PEDOT-PSS-based electrochemical sensor toward DA oxidation. Five ratios; i.e., 1:1, 2:1, 3:1, 1:2, and 1:3 of OMC: PEDOT-PSS by volume were optimized and evaluated in terms of sensitivity of DA detection between 2.0 to 100 μmol L^−1^, using differential pulse voltammetry (DPV). As illustrated in Figure 4, the optimal ratio of OMC and PEDOT-PSS composite was 2:1, which exhibited the highest sensitivity, indicating that the amount of OMC helped to enhance the catalytic activity. In contrast, an excessive amount of OMC in the OMC-PEDOT-PSS composite could lead to a higher background current, resulting in a decrease in the sensitivity [31]. Hence, the composite of OMC and PEDOT-PSS at 2:1 ratio was chosen to modify on a SPCE for an on-chip analysis experiment.

### 3.4. Interference Study

**Figure 5:**
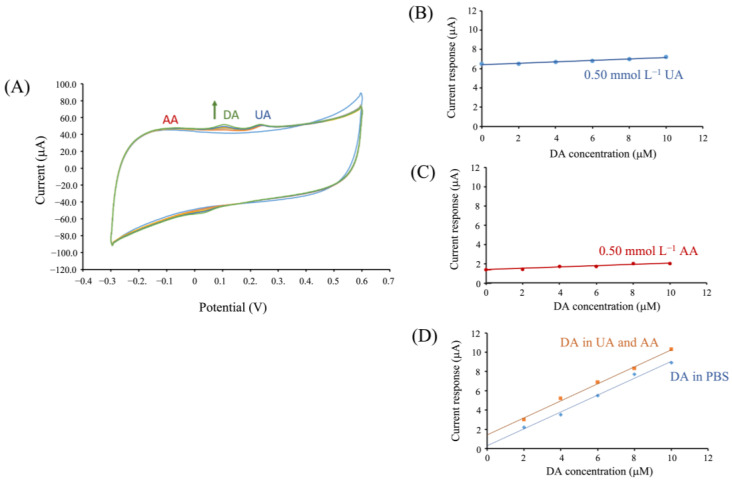
(**A**) Cyclic voltammograms in 0.10 mol L^−1^ PBS at pH 7.40 containing 0.50 mmol L^−1^ AA, 0.50 mmol L^−1^ UA, and the series of DA concentrations from 2.0 to 10 μmol L^−1^ DA at the OMC-PEDOT-PSS/SPCE. (**B**,**C**) The graphs show the mutual effect of UA, and AA plotted the change in current response of each substance versus DA concentration, respectively. (**D**) The graph compares between dopamine signal in PBS and in both UA and AA interferences.

The interference of DA detection for OMC-PEDOT-PSS/SPCE was evaluated by determining the interfering substances (AA and UA as coexisting in real biological samples), which could be oxidized at a close oxidation potential of DA. Cyclic voltammograms were obtained at a scan rate of 0.050 V s^−1^. The results (Figure 5A) showed that the oxidation peaks of AA, DA, and UA at −0.050 V, 0.17 V, and 0.30 V, respectively, were clearly separated. The mutual effect at the high concentrations of UA and AA indicated that both current responses were slightly increased when adding the series of DA concentrations (Figure 5B,C). At low concentrations of DA, the current signal of excessive AA was almost absent (at −0.050 V). This indicated that the DA detection at an OMC-PEDOT-PSS/SPCE coating, with Nafion as a protective film, was not disturbed by the AA. The modified sensor could contribute to eliminating the influence of AA, because of the negative charge of Nafion expelling the negatively charged AA [45]. However, when increasing DA concentrations, an increase in the current background was noticed. In the case of UA interference, although the oxidation current signal of UA at the developed sensor was present (at 0.30 V), its oxidation peak potential was higher (more positive) than that of DA [46]. Consequently, the DA detection using amperometry was not disturbed by the effect of UA. In addition, the sensitivity of DA signal did not change when compared between DA in PBS and interference (UA and AA). So, this sensor can protect interference and the effect of DA signal in interference also (Figure 5D).

When considering the peak-to-peak separation, the result showed that the oxidation peak separation of DA from AA was 220 mV, and from UA was 130 mV in the presence of 0.50 mmol L^−1^ AA and 0.50 mmol L^−1^ UA, respectively. This suggests that the developed OMC-PEDOT-PSS composite sensor enables the simultaneous detection of the three substances without any intermolecular effects. Therefore, this indicates that our developed OMC-PEDOT-PSS sensor exhibits an efficient electrocatalytic activity towards DA oxidation.

### 3.5. Dopamine Determination Using a Microfluidic Platform

**Figure 6:**
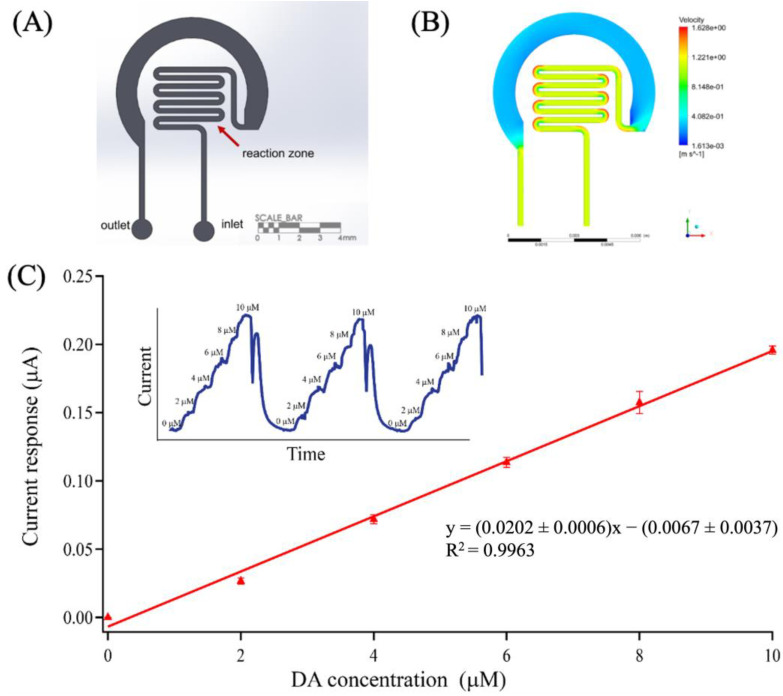
(**A**) Design of a serpentine microfluidic channel used for integrating on OMC-PEDOT-PSS/SPCE; showing the position of the inlet, the outlet and the reaction zone. (**B**) Top view: velocity profile of a serpentine microfluidic chip showing flow behaviors in serpentine microfluidic chip, using ANSYS Fluent CFX. (**C**) Calibration plot obtained using an autocalibration microfluidic sensor platform. The inset showed an example of continuous calibration for three cycles using 5-point standard addition at a constant 2.0 μL min^−1^ total flow rate. The reduction in the current signal after the auto-calibration is complete flow dopamine from the syringe. The next step is filling the PBS solution in the syringe and flow the PBS solution into the microfluidic channel for access to the base line. However, there are some solutions of dopamine in the tube; thus, the current signal increased and decreased again as the dopamine solution was leached.

The microfluidic-based OMC-PEDOT-PSS sensor system was investigated for continuous DA detection using an in-house autocalibration microfluidic system. The proposed pattern of a serpentine microfluidic channel (Figure 6A) was first analyzed for its flow behavior by simulation using ANSYS Fluent (2019 R1). A flow rate of 2.0 μL min^−1^ (typically used for collecting samples using microdialysis) [1] was set for the simulation study. The resulting velocity profile is presented in Figure 6B, showing a continuous flow through the serpentine microchannel with a homogenous velocity in the reaction zone (Reynolds number, Re = 0.0014 indicating laminar flow).

To evaluate the serpentine microfluidic OMC-PEDOT-PSS/SPCE platform using an in-house automated calibration board for DA determination, amperometric detection was performed by applying a constant potential of +0.20 V. As shown in Figure 6C (inset), the excellent repeatability of the same sensor was tested three times in the range from 2.0 to 10.0 μmol L^−1^ DA standard solution. The sensitivity of our microfluidic-based OMC-PEDOT-PSS sensor was 20.2 ± 0.6 μA μmol L^−1^, and the limit of detection (LOD) was calculated to be 21.6 ± 0.002 nmol L^−1^ (3SD_blank_/sensitivity) with the confidence level of 99%. This suggested that the serpentine microfluidic-based OMC-PEDOT-PSS/SPCE system could provide well mixing and continuous flow through the reaction zone. In addition, the serpentine microfluidic-based OMC-PEDOT-PSS/SPCE could contribute to minimizing the electrochemical fouling of the DA sensor on the working electrode surface, compared to the response signals of the circle microfluidic-based OMC-PEDOT-PSS/SPCE (Figure S1).

**Figure 7:**
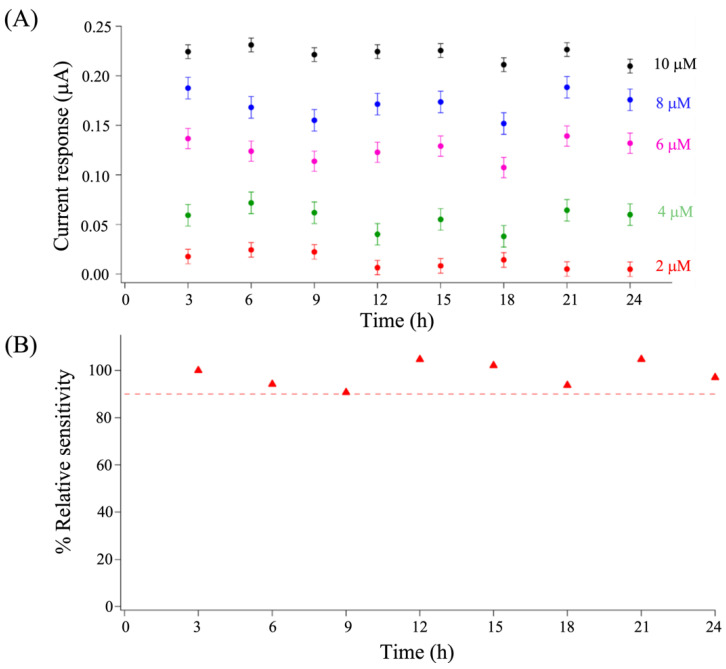
Stability of the serpentine microfluidic OMC-PEDOT-PSS/SPCE platform. (**A**) Current responses in each DA concentration. The plot was obtained when continuous monitoring of DA for 24 h (recorded every 3 h) was carried out in a range of 2 to 10 μmol L^−1^ DA concentration at a constant 2.0 μL min^−1^ total flow rate. (**B**) Plotting relative sensitivity of the developed sensor for a period of 24 h.

To show the applicability of this sensor platform for continuous DA detection, the stability of the serpentine microfluidic-based OMC-PEDOT-PSS/SPCE was tested by determining different DA concentrations from 2.0 to 10 μmol L^−1^ for 24 h. Figure 7A shows the electrochemical responses of the 5-point calibration concentrations every 3 h. The sensor stability was examined further from its relative sensitivity (Figure 7B), and it was observed to be more than 90% [47] over time. This could be explained in that the serpentine microfluidic-based OMC-PEDOT-PSS/SPCE was exposed to a small volume of DA for a short period under continuous flow, so the DA oxidation products could be washed out. Hence, our developed microfluidic OMC-PEDOT-PSS/SPCE platform exhibits excellent stability for continuous monitoring of dopamine, even at room temperature.

The analytical performance of the microfluidic-based OMC-PEDOT-PSS/SPCE as a dopamine sensor was compared with other electrochemical sensors for dopamine detection, as shown in Table 1. Our developed sensor system exhibited the highest sensitivity with a low sample volume. Although the limit of detection was not low as in some similar work [23], our sensor offered better performance than other cited reports.

## 4. Conclusions

In this work, we present the development of a microfluidic-based electrochemical sensor for dopamine detection under a continuous flow system. Under optimum conditions, the OMC-PEDOT-PSS modified SPCE at 2:1 ratio was chosen for integrating with a PDMS-based serpentine microfluidic chip, as it exhibited the greatest sensitivity to dopamine. The serpentine microfluidic-based OMC-PEDOT-PSS/SPCE platform was successfully developed for DA amperometric detection using an in-house computer-controlled calibration board. The developed dopamine sensor exhibited a sensitivity of 20.2 ± 0.6 μA μmol L^−1^, and a low detection limit of 21.6 ± 0.002 nmol L^−1^, with high selectivity. Significantly, the developed DA sensor platform could reduce the effect of electrochemical sensor fouling, which provided excellent stability for over 24 h under continuous DA detection. In addition, this system offers an easy way for tracking the sensor performances with simplified operation, as there was no need for trained personnel through our in-house autocalibration system. Future studies will be focused on the DA monitoring in real samples, such as cerebrospinal fluid, and it may be possible to make the entire detection system more compact and portable. Hence, this novel platform could be a powerful tool that allows us to monitor dynamic chemical changes of dopamine in real-time, showing a great possibility for human clinical applications.

## Figures and Tables

**Table 1:** A comparison of the performance of microfluidic based electrochemical sensors for dopamine detection.

Electrode Material	Detection Method	Limit of Detection (nM)	Sensitivity (μA μM^−1^)	Working Volume (μL)	Ref
CFμE	FSCV	NR	0.013	50	[48]
AuμE	Amperometry	0.1	3.53	2.40	[23]
CAuNE	Amperometry	5800	0.034	NR	[49]
MPA/Au µE	Amperometry	74	NR	0.120	[50]
OMC-PEDOT-PSS/SPCE	Amperometry	21.6	20.2	0.675	This work

Note: CFμE: carbon-fibre microelectrode, FSCV: fast scan cyclic voltammetry, AuμE: gold microelectrode, CAuNE: cylindrical Au nanoelectrode, MPA/Au μE: mercaptopropionic acid-modified Au microelectrode, NR: no report.

## Data Availability

Data available on request from the authors.

## Supplementary Materials

The following supporting information can be downloaded at: https://www.mdpi.com/article/10.3390/bios13010068/s1, Figure S1: Comparison the analytical performances of between circle and serpentine microchannel for dopamine continuous detection testing at 2, 4, 6, 8 and 10 μM. As compared to the serpentine microfluidic sensor (red) when testing continuous DA detection using in-house auto-mated calibration platform, the circle microfluidic based OMC-PEDOT-PSS/SPCE (blue) showed less sensitivity and also poorer stability. It may be due to electrode fouling; Figure S2: Velocity profile showing in a circle based microfluidic chip using ANSYS Fluent CFX. The circle pattern of microchannel for inte-grating with SPCE is consisted of one inlet, one outlet, and a reaction zone, which the channel dimension was 100 μm in depth, 250 μm in width, and 10.4 mm^2^ for circle area as a reaction zone (2.0 mm in radius). For setting flow rate of solution is 2 μL/min, at the circle region it showed that the velocity of solution was low, leading to previous DA standard solution remaining in this area. Moreover, it was easy to generate bubbles during the experiment consequently, it was difficult to flush out those bubbles because the velocity of solution was not enough for washing.
