# Supplementary material for: A Novel Microfluidic-Based OMC-PEDOT-PSS Composite Electrochemical Sensor for Continuous Dopamine Monitoring
Source: Biosensors (Basel). 2022 Dec 31;13(1):68. doi: 10.3390/bios13010068 (PMC9855352; doi:10.3390/bios13010068)
Supplement: Supplementary file 1 [file biosensors-13-00068-s001.zip › biosensors-2084942-supplementary.pdf]

biosensors

MDPI

Supplementary Data

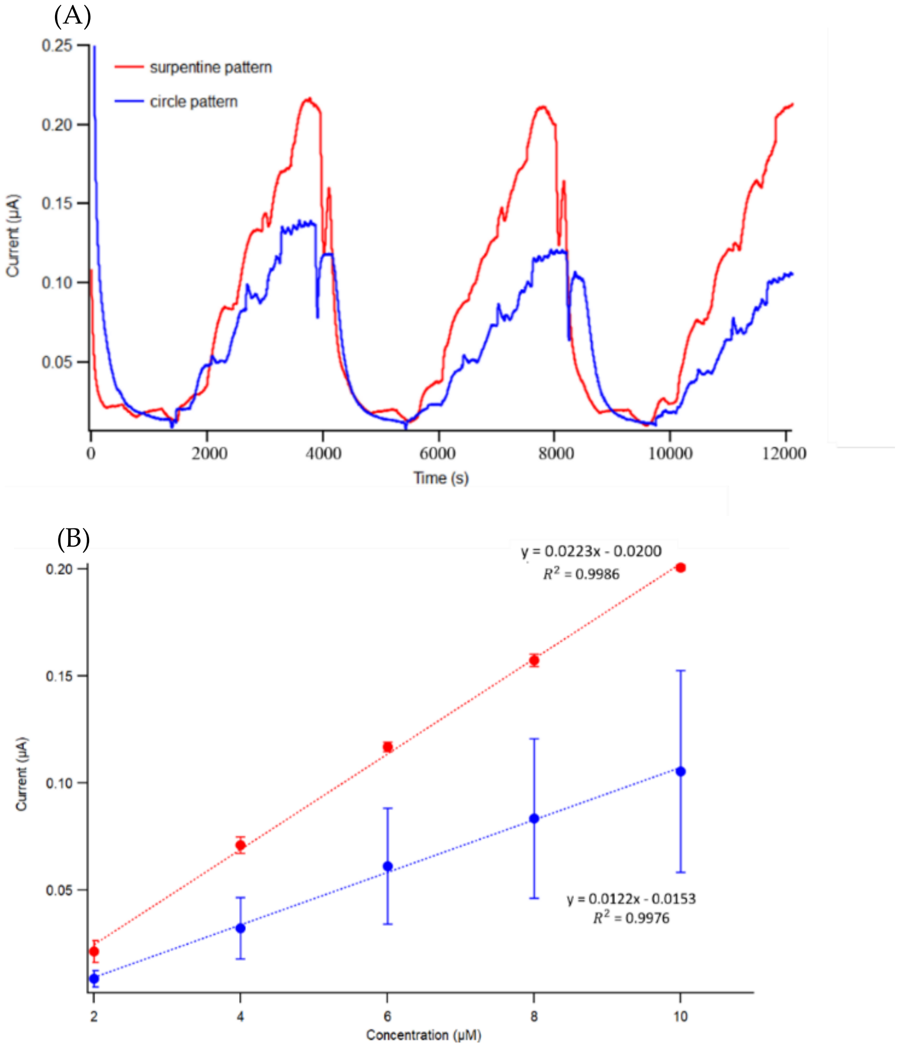

Figure S1. Comparison the analytical performances of between circle and serpentine microchannel for dopamine continuous detection testing at 2, 4, 6, 8 and 10 µM. As compared to the serpentine microfluidic sensor (red) when testing continuous DA detection using in-house auto-mated calibration platform, the circle microfluidic based OMC-PEDOT-PSS/SPCE (blue) showed less sensitivity and also poorer stability. It may be due to electrode fouling.

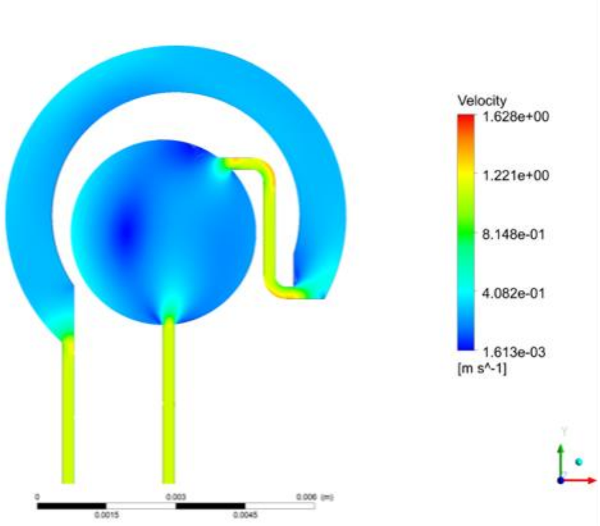

Figure S2. Velocity profile showing in a circle based microfluidic chip using ANSYS Fluent CFX. The circle pattern of microchannel for integrating with SPCE is consisted of one inlet, one outlet, and a reaction zone, which the channel dimension was 100 µm in depth, 250 µm in width, and 10.4 mm^2 for circle area as a reaction zone (2.0 mm in radius). For setting flow rate of solution is 2 µL/min, at the circle region it showed that the velocity of solution was low, leading to previous DA standard solution remaining in this area. Moreover, it was easy to generate bubbles during the experiment consequently, it was difficult to flush out those bubbles because the velocity of solution was not enough for washing.
